# Development of an electronic health message system to support recovery after stroke: Inspiring Virtual Enabled Resources following Vascular Events (iVERVE)

**DOI:** 10.2147/PPA.S154581

**Published:** 2018-07-11

**Authors:** Dominique A Cadilhac, Doreen Busingye, Jonathan C Li, Nadine E Andrew, Monique F Kilkenny, Amanda G Thrift, Vincent Thijs, Maree L Hackett, Ian Kneebone, Natasha A Lannin, Alana Stewart, Ida Dempsey, Jan Cameron

**Affiliations:** 1 Stroke and Ageing Research, Department of Medicine, School of Clinical Sciences at Monash Health, Monash University, Melbourne, VIC, Australia, dominique.cadilhac@monash.edu; 2 Stroke Division, Florey Institute of Neuroscience and Mental Health, The University of Melbourne, Melbourne, VIC, Australia, dominique.cadilhac@monash.edu; 3 Department of Electrical and Computer Systems Engineering, Monash University, Melbourne, VIC, Australia; 4 Department of Medicine, Central Clinical School, Monash University, Melbourne, VIC, Australia; 5 Department of Neurology, Austin Health, Melbourne, VIC, Australia; 6 The George Institute for Global Health, University of New South Wales, Sydney, NSW, Australia; 7 Faculty of Health and Wellbeing, University of Central Lancashire, Preston, UK; 8 Graduate School of Health, University of Technology Sydney, Ultimo, NSW, Australia; 9 College of Science, Health and Engineering, School of Allied Health, La Trobe University, Melbourne, VIC, Australia; 10 Occupational Therapy Department, Alfred Health, Melbourne, VIC, Australia; 11 Stroke Foundation, Melbourne, VIC, Australia; 12 Consumer Representative, Melbourne, VIC, Australia; 13 Australian Centre for Heart Health, Royal Melbourne Hospital, Melbourne, VIC, Australia

**Keywords:** stroke, e-health, self-management, health promotion, secondary prevention

## Abstract

**Purpose:**

Worldwide, stroke is a leading cause of disease burden. Many survivors have unmet needs after discharge from hospital. Electronic communication technology to support post-discharge care has not been used for patients with stroke. In this paper, we describe the development of a novel electronic messaging system designed for survivors of stroke to support their goals of recovery and secondary prevention after hospital discharge.

**Participants and methods:**

This was a formative evaluation study. The design was informed by a literature search, existing data from survivors of stroke, and behavior change theories. We established two working groups; one for developing the electronic infrastructure and the other (comprising researchers, clinical experts and consumer representatives) for establishing the patient-centered program. Following agreement on the categories for the goal-setting menu, we drafted relevant messages to support and educate patients. These messages were then independently reviewed by multiple topic experts. Concurrently, we established an online database to capture participant characteristics and then integrated this database with a purpose-built messaging system. We conducted alpha testing of the approach using the first 60 messages.

**Results:**

The initial goal-setting menu comprised 26 subcategories. Following expert review, another 8 goal subcategories were added to the secondary prevention category: managing cholesterol; smoking; physical activity; alcohol consumption; weight management; medication management; access to health professionals, and self-care. Initially, 455 health messages were created by members of working group 2. Following refinement and mapping to different goals by the project team, 980 health messages across the health goals and 69 general motivational messages were formulated. Seventeen independent reviewers assessed the messages and suggested adding 73 messages and removing 16 (2%). Overall, 1,233 messages (18 administrative, 69 general motivation and 1,146 health-related) were created.

**Conclusion:**

This novel electronic self-management support system is ready to be pilot tested in a randomized controlled trial in patients with stroke.

## Introduction

Globally, stroke is the third most common cause of disability with >10 million strokes occurring annually and almost 30 million people living with a history of stroke.^1^,^2^ For most survivors, integrating back into the community and managing the consequences of stroke is difficult. Survivors of stroke liken the experience of going home from hospital to “falling into a black hole”.^3^ This situation arises because stroke can have a profound impact on quality of life, with approximately one-quarter of survivors reporting their situations as equivalent to, or worse than, death.^4^ Factors that complicate return to the community after stroke include the presence of physical disability, cognitive changes, loss of employment, inability to participate in pre-stroke activities, social isolation, anxiety and depression.^5^ Survivors also have an increased risk of vascular events,^6^ and many have modifiable risk factors that are poorly managed.

Despite clear recommendations for discharge preparation in national guidelines for stroke care,^7^ discharge care is poorly implemented in hospitals. Only half of the patients with stroke receive a formal care plan at the time of discharge or lifestyle education to support moderating their future risk of vascular disease.^8^ In an Australian survey of 765 survivors one or more years following stroke, 84% reported unmet recovery and secondary prevention needs, particularly in aspects of health such as cognition, memory, emotion and fatigue.^9^ The short length of hospital stay of ~5 days in Australia^10^ may contribute to the lack of adequate self-management tools and support provided to patients in hospital. Approximately 40% of survivors of stroke, and >60% of their caregivers, report being dissatisfied with the information they received while in hospital.^11^ Patients also want to be actively involved in setting their goals to positively influence their recovery.^12^ However, there are often mismatches between what clinicians and patients consider to be patient-centered goal setting.^13^

Comprehensive management and goal setting after discharge is aimed at addressing the self-management needs of each patient, including their return to usual activities in their home environment, addressing personal concerns and mini-mizing the risk of future stroke.^14^ However, providing these patients with the necessary support to continue with their recovery and secondary prevention once they have been discharged home is challenging, particularly in those with limited mobility or who live a long distance from hospitals/services. Simple, innovative and cost-effective strategies could be used to help survivors attain their secondary prevention goals, adjust to their altered functional and emotional abilities, and promote community participation.^7^ Because of the exponential increase in use of electronic devices, for example, mobile phones, tablets and personal computers in the community, these electronic communication technologies could be used to promote changes in lifestyle behavior and self-management.

Although short message service (SMS) and internet-based programs have been trialled in several populations^15^ their use in survivors of stroke is less common and limited to improving medication adherence or reducing depression in men.^16^,^17^ Given the broad impact stroke has on survivors, the aim of our project was to develop a comprehensive, electronic self-management support program designed for attaining patient-centered goals.

## Materials and methods

The Inspiring Virtual Enabled Resources following Vascular Events (iVERVE) pilot establishment project was achieved through collaboration between the Faculty of Medicine, Nursing and Health Sciences and the Faculty of Engineering at Monash University, Australia. Leading academics with an interest in self-management programs for chronic conditions from other institutions were also involved. We undertook a literature review of electronic health interventions to ensure our program design was based on the latest evidence. We then used a formative program evaluation to ensure the system was robust. Formative evaluation enables development and modification of a program within a feedback loop to promote successful future implementation.^18^

**Figure 1:**
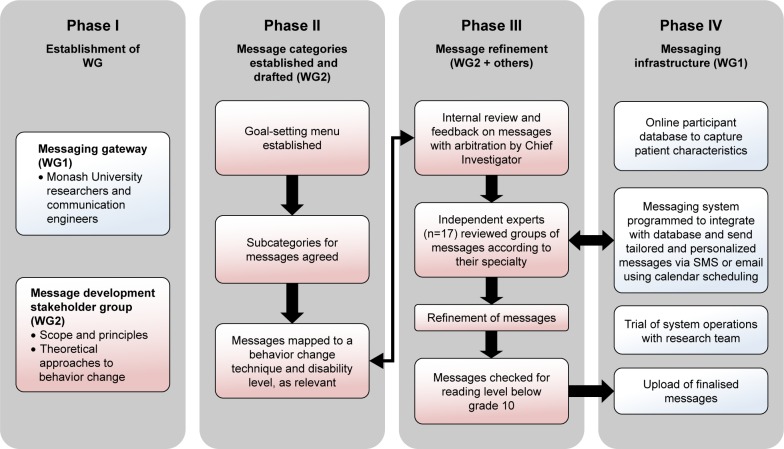
Project phases and activities. **Abbreviation:** WG, working group.

The iVERVE pilot project was established in four phases. Phase I involved establishment of two working groups: working group 1 for the technical application/infrastructure; and working group 2 for the content and refinement of messages. In Phase II, we developed a goal-setting menu comprising broad categories of recovery and secondary prevention categories. These categories were then subcategorized to enable the formulation of messages. Phase III comprised refinement of messages, using internal and external review, while Phase IV comprised establishing and trialling the concurrently developed iVERVE messaging system (Figure 1). Further detail is provided in the following sections.

The working groups were comprised of investigators based on their relevant expertise, clinicians, project staff and consumer representatives or engineers, as relevant. The lead principal investigator (DAC), project coordinators (JC and DB), and MFK, AGT and NEA from Monash University were members of both working groups to ensure that concurrent work was complementary and that the experience gained in one area could be used to inform the other area.

Ethical approval for this project was provided by Monash University Human Research Ethics Committee (CF16/1920-2016000979). As this was early development phase research, the testing of the system by researchers was considered to not require written informed consent.

### Messaging infrastructure development and testing (working group 1)

Telecommunications engineers from the Faculty of Engineering at Monash University were responsible for creating the specified electronic messaging system and web interface. The system was developed during several meetings to fulfil our requirements to incorporate scheduling, personalization (ie, ability to include first, last name or preferred names), and tailoring of the support messages to align with a patient’s disability level and their specified health and recovery goals.

A participant database was created to enter patient demographic information and severity of stroke. Information obtained from patients about their preferred scheduling frequency or mode of communication (eg, by SMS or email) was also incorporated into this database. The messaging system was programmed for integration with the database used for storing a much larger range of variables for each participant. This was to avoid double data entry of patient information. For future use in clinical trials, the participant database was created using Research Electronic Data Capture (REDCap) tool hosted at Monash University. REDCap is a secure, web-based application designed to support data capture for research studies, providing: 1) an intuitive interface for validated data entry; 2) audit trails for tracking data manipulation and export procedures; 3) automated export procedures for seamless data downloads to common statistical packages; and 4) procedures for importing data from external sources.^19^ We designed the database to be partitioned for different users, whereby only the project coordinator could access all information. The system also incorporates an inbuilt electronic randomization program.

Messages were bundled, with the iVERVE messaging system programmed to randomly select a message from a themed predetermined bundle until all messages from that bundle had been sent at least once. This provides a large range of message options for each goal that can be pre-assigned for each patient for each of their specified goals. This process was developed to reduce the labor intensity in pre-assigning messages for each specific personalized goal. Individual messages within the bundle were categorized into those more appropriate during the first weeks of participation (ie, “early”) and for more advanced stages of goal attainment (“late”; “Theoretical basis for message development” section). The system was also designed so that electronic messages could be sent via email if this was the participant’s preferred mode of receiving messages. The ability to delete, edit or add messages was also built into the system.

A dedicated phone number was purchased through a commercial bulk SMS provider. This ensured that patients would only receive messages from a unique iVERVE phone number. To ensure appropriate and efficient documentation of activity, the system was designed with an automated log record of all electronic messages sent to individual participants (date, time, copy of message), including any that had failed to send. Bi-directional communication was required to permit simple responses (eg, Yes or No) to incoming messages when requested, as well as the option to send a “STOP” message to opt out of receiving some or all the electronic messages.

Alpha testing of the system, by the project team, was conducted throughout the various development stages, similar to an agile method.^20^ Once an initial 60 messages had been developed, they were uploaded onto the electronic website and members of the research team were scheduled to receive SMS or email messages for various health/recovery goals. Revisions were made following feedback on the wording of messages, spelling errors, or failure of the shortened URL Web links. All other technical issues were also recorded and rectified.

### Development of the goal-setting menu and self-management support messages (working group 2)

We established a working group consisting of clinicians, behavioral and social scientists, psychologists, and epidemiologists with experience in conducting research using SMS or e-health interventions in stroke or similar chronic conditions.^21^–^23^ A representative from the Stroke Foundation and a consumer (ie, survivor of stroke) also contributed to this working group. Terms of reference were established, and monthly or bi-monthly meetings were arranged via teleconference or in person.

In the first stage, consensus agreement from the working group was sought to provide feedback on a draft standardized goal-setting menu (Table 1). Setting goals for recovery based on “needs” can be highly effective and use of a standardized goal-setting menu can reduce subjectivity, ensure goals are measurable, and reduce the time taken to set goals.^24^ For this aspect, we used existing data from observational studies to ensure we included all relevant areas important for achieving a patient-centered approach to meeting identified recovery or secondary prevention goals. In particular, we relied on data from the Australian Stroke Survivor and Carer Needs assessment survey that was based on a similar survey conducted in the UK.^9^,^25^ In the Australian version, 88% of the N=765 respondents felt that the scope of the survey questions covered their needs.^26^

The components of the draft goal-setting menu used in this project were then mapped to the International Classification of Functioning Disability and Health (health and body functions, activities and participation, environment)^27^ by NEA within the context of unmet needs^9^ to ensure that all relevant aspects of recovery were covered. Secondary prevention of future stroke is very important to survivors and forms part of best-practice clinical guidelines.^7^ Therefore, a secondary prevention category was added to the menu.

Following consensus on the goal-setting menu and subcategories for each main goal, this menu was used as the basis for developing the support/educational messages. Members of the working group volunteered to develop preliminary health messages selected from a subcategory that matched their area of expertise. As part of this process, contributors were asked to draw upon reputable and current clinical guidelines for management of stroke or prevention of cardiovascular disease and provide appropriate and trusted Web links of the source information.

Contributors were asked to follow set criteria for developing messages. Messages were required to be applicable to people of any age or sex, cater for different levels of impairment (disability), and allow for progression over a 12-week period of support. The maximum message length was set at 160 characters to allow messages to be sent as a single SMS. Use of commonly accepted abbreviations was permitted. Messages were also required to be developed using plain language that could be understood by someone with a reading level below grade ten (equivalent to a reading level of people aged 15–16 years). Each working group member was asked to develop a minimum of 8 messages for their subcategory. This was to enable a sufficient number of messages to be produced for a 12-week program of support.

### Theoretical basis for message development

The messages targeted at attaining stroke recovery goals or maintaining motivation for secondary prevention goals were designed on the basis of 21 behavior change techniques (BCTs) that are underpinned by up to 7 behavior change theories (Table 2). This approach was used because of the evidence that attainment of individual goals is more likely when the messages are targeted at specific behavior change constructs.^28^,^29^ Specifically, working group members were asked to match each message to a specific BCT as reported by Abraham and Michie et al^29^ (Table 2) and adapted by Redfern et al.^22^ This taxonomy of BCTs has been developed to standardize and characterize the content of behavior change interventions that have been linked to mediating processes informed by the relevant behavior change theories. Similar to Redfern et al,^22^ messages were developed with a positive reinforcement stance. This has been shown to be more effective in producing lasting behavior change. For example, messages targeted positive reinforcement in the form of social reward such as encouragement, feelings of mastery from goal attainment, or experiencing benefits from improvements in health (Table 2).

To enable logical scheduling, messages were developed and grouped into early (contemplation/preparation) and late (action/maintenance) stages based on the Transtheoretical model of health behavior change (TTM), as outlined above. We acknowledge the relationships associated with behavior are complex and may be non-linear. However, the TTM is particularly helpful when developing interventions that address multiple behaviors within one program. The TTM has been used successfully in several settings for behavior change relating to stroke and cardiovascular risk reduction, whereby specific strategies have been applied to address the individual’s readiness to change.^30^–^33^ Considering that individuals who receive the self-management messages will have set their own health and recovery goals, they will have passed the pre-contemplation stage. On this basis, members of the working group were asked to map each message to either the contemplation/preparation, or action/maintenance phase.

To assist in tailoring messages to individual ability, potential barriers to self-management were assessed based on application of the Theoretical Domains Framework.^34^,^35^ These domains provide wide coverage of potential barriers to achieving behavior change and include: knowledge; skills; social role and identity; beliefs about capabilities; optimism; beliefs about consequences; reinforcement; intentions; goals; memory, attention and decision processes; environmental context and resources; social influences; emotion; and behavioral regulation. The working group members were asked to consider developing messages that addressed these potential barriers to self-management. For example, to overcome lack of knowledge, messages were developed with links to reputable websites from where more information could be accessed regarding a specific behavior or goal.

Where appropriate, messages were further categorized to cover a range of disability/functional levels, for example, slight or moderate disability and severe disability. In this manner, health messages could be tailored to the individual’s level of disability on the basis of using the modified Rankin scale, a disability measure commonly used in stroke populations. Finally, Google URL (https://goo.gl/) was used to create shortened Web links.

### Internal review of health messages

Two investigators (DB and JC) reviewed all the messages returned by members of the working group. As bundles of messages were completed, they were reviewed by the principal investigator (DAC) who made editorial changes, suggested new messages, or wrote additional messages, when required. Where the character limit permitted, it was decided that all messages would be personalized with a first name.

### Validation of messages via independent review

Health professionals and stroke researchers not contributing to iVERVE working groups were identified and invited to independently review the initial set of messages based on their field of expertise. The messages were sent via email with instructions on the review process. Specifically, they were asked to review the appropriateness and wording of messages, the stage(s) of change classification and, where relevant, the level of (dis)ability targeted for each message. They were also asked to consider providing additional messages appropriate to their allocated topic when they considered that an important message had been overlooked.

### Confirmation of final bank of messages

Feedback from the independent panel was initially reviewed by 2 investigators (JC and DB) and the principal investigator (DAC) arbitrated to achieve consensus, as required. The final-ized messages were then checked for reading level, below grade 10, using an online software (http://www.webpagefx. com/tools/read-able/). The authors of this software consider that if the text has an average grade level of about 10, it should be easily understood by people aged 15–16 years.

## Results

The initial draft goal setting menu based on the Australian Stroke Survivor Needs Survey items, consisted of 26 subcategories. Following input from the expert panel, the household chores subcategory was further categorized to inside chores and outside chores, whereas the subcategories of emotions, concentration and memory were combined into a single subcategory. Eight additional subcategories were identified by the panel. The additional goals were: managing cholesterol; smoking; physical activity; alcohol consumption; weight management; medication management; access to health professionals, for example, General Practitioner; and self care (eg activities of daily living). Overall, this resulted in 32 subcategories for the 4 main categories of the goal-setting menu (Table 1).

**Figure 2:**
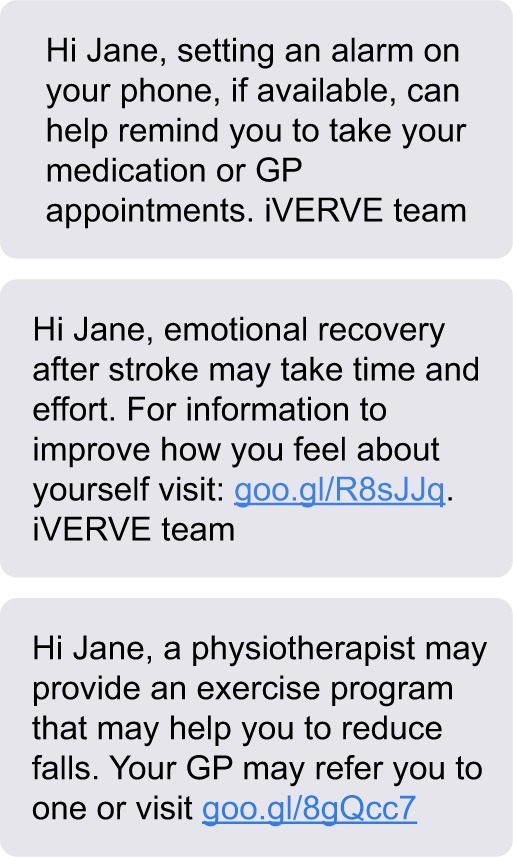
Sample messages for a fictitious person. **Abbreviations:** iVERVE, Inspiring Virtual Enabled Resources following Vascular Events; GP, General Practitioner.

For the initial stage of message development, 455 health messages were created by members of working group 2 (Figure 2 shows examples of messages). These were then refined by project staff and additional messages created based on suggestions for addressing gaps that had been identified by the working group. Messages were then mapped to other goals and duplicated, where appropriate. For example, the message “Activity burns calories and helps maintain weight. Climbing stairs, parking further away, or walking to the office add up quickly to 30 min a day” was suitable for a weight loss and physical activity goal. This internal review process resulted in 980 health messages related to health and recovery goals and 69 general motivational messages.

**Figure 3:**
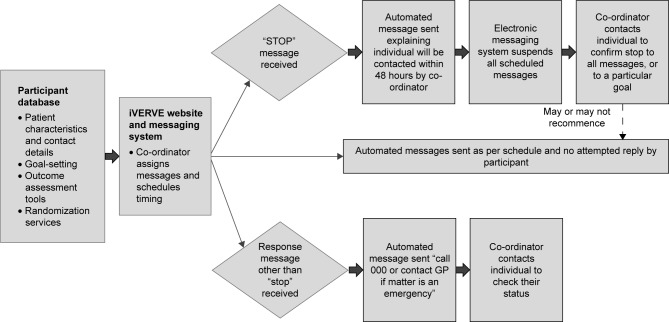
Messaging system and responses to messages. **Abbreviations:** iVERVE, Inspiring Virtual Enabled Resources following Vascular Events; GP, General Practitioner.

During alpha testing of the messaging system, we identified that we needed to program an automated message as a response to all “STOP” messages. This standard automatic message from the iVERVE project staff would indicate to the participant that they will be contacted within 48 hours regarding their request to STOP. In this circumstance, the system would temporarily suspend all other messages that had been scheduled for this participant until the iVERVE team clarified with them the reason for wanting to stop messages (Figure 3). In the event that a message other than “Yes/No/STOP” was to be received, an automated message was programmed to be sent with instructions to “Call 000 or contact a GP, if the matter is an emergency”. We also identified that several other administrative messages would be needed, including a “welcome” message.

### Independent review

Seventeen independent reviewers, including health professionals and researchers, agreed to review bundles of messages. The independent reviewers disagreed with only 16 of the messages (2%) and suggested 73 additional messages (Table 3). Following the independent review process, all health and motivational messages were assessed for level of readability, whereby 96% (1,097/1,215) met our objective with a reading level of Grade 10 or below. Moreover, 79% were at a readability level of Grade 8 and below (equivalent to a reading level of adolescents aged 13–14 years).

Overall, we developed a final set of 1,233 messages (1,146 health or recovery-related, 18 administrative and 69 general motivational messages), with several duplicated across goals where relevant (Table 4). Shortened URL Web links were created on 27% (333/1,215) of the non-administrative messages using the Google URL: https://goo.gl/. In addition, more than one-third of the developed messages could be personalized.

## Discussion

Our findings add to the limited literature on the process of developing an electronic self-management support messaging program to promote health behavior change. Most importantly, we have developed a bank of health messages and infrastructure that can be used to promote secondary prevention and recovery from stroke. Most messages were designed for 1-way communication. However, some 2-way messages were included as this format has been shown to encourage greater patient engagement.^15^ Combined with the fact that messages were informed by behavioral theories, these bidirectional messages may enhance the potential for successful behavior change in the targeted population.

Our iVERVE intervention, which comprises a goal-setting menu that can be used by clinicians with aligned support messages, will address many of the aforementioned barriers to discharge planning such as poor communication from clinicians with patients by augmenting the current support offered to patients in the post-discharge period for up to 12 weeks. Our plan now is to test the effectiveness potential of this novel intervention for stroke by undertaking Phase I (proof-of-concept), II (acceptability and feasibility) and III (powered for health outcome effects) randomized controlled trials. In these trials, we will recruit patients with stroke and use the goal-setting menu to standardize and pri-oritize mutually agreed recovery and secondary prevention goals. Patient data and agreed goals will be entered into an electronic Case Report Form (eCRF), which will automatically prepopulate the iVERVE system. A clinician researcher can then link appropriate bundles of messages to each of the goals for a specified period (eg, 12 weeks). It is our intention to contact patients in the intervention group at least 3 times per week; the maximum number of contacts will be daily. Messages will be scheduled according to the priority of the goals, and stage of readiness for change, with a focus on only 1 goal in week 1 and then gradual introduction of other goals. This staggered approach was chosen to avoid overwhelming participants. In addition, 1 or 2 administrative or general motivational messages will be sent each week. This future work will provide essential information for determining the practicalities of implementing this new intervention and its potential effectiveness (eg, fewer readmissions to hospital, increased self-efficacy, and persistence with secondary prevention) and cost-effectiveness.

None of the prior research on SMS and email messaging support has offered a comprehensive solution to addressing the complexity of living with stroke. Appropriately designed trials in stroke are urgently needed.

The large and comprehensively developed bank of iVERVE messages is, to our knowledge, the first of its kind and is of great importance in interventions targeted at secondary prevention and recovery in survivors of stroke. Additionally, the novelty of our study is that the health messages have been specifically designed with a patient-centered focus to promote attainment of individual patient goals. An advantage of using text messages as the basis of interventions is that they can be used with almost all mobile phones, can be personalized, and take advantage of the widespread use of texting in most populations globally.^36^,^37^ The uptake of this technology in survivors of stroke is likely to increase in the future with the predicted increases in use of technology in older populations.

A robust process was used in the development of our program and messages were based on a range of complementary theoretical frameworks. These messages were mapped to various BCTs to promote beneficial behavior. Additionally, messages were based on evidence-based information and Stroke Clinical Guidelines^7^ since we did not seek to recreate reliable education material or content. In particular, a main principle was that the strategies or suggestions we provided would encourage and empower patients to seek out the information as part of their self-management.

Another strength is the ability to use SMS or email to communicate with participants. While availability of Internet-based resources does not necessarily mean they will be used, uptake of technology is greater when it is based on tools/devices already in everyday use,^38^ and there is evidence of willingness to learn new technologies and skills.^36^ In Australia, in 2016, 85% of the total population used the Internet.^39^ Furthermore, 79% of adults aged >65 years used the Internet, with most (85%) accessing it daily, and 74% owning a mobile phone.^39^ Therefore, participants who have suffered a stroke (generally aged over 65 years) are likely to have access to, and use, technology.

A limitation of this study is that messages developed were written in English and further research is needed to develop text messages that are suitable for culturally and linguistically diverse groups, or those with communication disorders. This limitation notwithstanding, a strength of our study is that messages were developed using evidence-based methods. Importantly, feedback from the independent review demonstrated essential consensus to the messages that we had developed with requests mainly being for minor changes.

## Conclusion

We have described the development of a novel electronic self-management support system designed for survivors of stroke after hospital discharge. The intervention addresses the full scope of potential needs for patients through a comprehensive goal-setting menu and includes an online database to capture participant characteristics with automatic integration of selected data into the purpose-built iVERVE messaging system. The platform has >1,200 electronic messages ready for testing for acceptability in clinical use. The messages were developed using evidence-based theory, clinical guidelines, and independent review. There is a growing body of evidence about the use and potential effectiveness of messaging-based interventions for reducing behavioral risk factors and improving cardiovascular disease management. The iVERVE intervention has the potential to promote secondary prevention and recovery in survivors of stroke and may help avoid unplanned readmissions. Since effectiveness data for e-Health are limited in stroke, future randomized controlled clinical trials are planned to use the iVERVE intervention.

## Figures and Tables

**Table 1:** Goal-setting menu developed for the iVERVE program

Major categories	Subcategories
Secondary prevention	Blood pressure Cholesterol^b^ Nutrition/diet Physical activity^b^ Smoking^b^ Alcohol consumption^b^ Weight management^b^ Medication adherence^b^
Health/body function^a^	Memory, concentration, cognition Emotions Falls Incontinence (urine and fecal) Speech and language Swallowing Pain Fatigue Mobility/walking Vision Involvement in decisions about treatment and care
Activities and participation^a^	Finances Return to work Spousal relationships Friend relationships Leisure activities Return to driving Use public transport Household chores (inside) Household chores (outside) Self-care (activities of daily living) for example, bathing, dressing, toileting^b^
Environment^a^	Access to information Access to health professionals for example, GP, allied health professionals^b^ Face-to-face or online support

**Notes:**

a Categories are consistent with the WHO International Classification of Functioning Disability and Health.^40^

b Additional items not identified from mapping data from the Australian Stroke Survivor Needs Survey.^9^

**Abbreviations:** iVERVE, Inspiring Virtual Enabled Resources following Vascular Events; GP, General Practitioner; WHO, World Health Organization.

**Table 2:** Behavior change techniques used to create health educational and motivational messages for the iVERVE program

Behavior change technique	Description	Example message
Provide information about behavior health link (IMB)	General information about behavioral risk and poor health outcomes	Do you know that taking blood pressure and cholesterol medicines reduces your risk of another stroke?
Provide information on consequences (IMB, TRA, TPB, SCogT)	Information about the benefits, costs, and consequences of action or inaction	Not only does physical activity reduce the risk of stroke but also reduces the risk of hypertension and diabetes.
Provide information about others’ approval (IMB, TRA, TRB)	What others think about the person’s behavior and whether others will approve or disapprove of any proposed behavior change	Participating in regular physical activity is important. Your friends and family think so too!
Prompt intention formation (IMB, TRA, TPB, SCogT)	To encourage forming a decision to act (ie, change behavior) or establish a goal to achieve behavior change	Joining a gym may be the motivation that you need to keep physically active. Sign up with a gym today.
Prompt barrier identification (SCogT)	Identify barriers to the desired behavior change and develop a plan to overcome barriers	Fatigue post stroke may prevent you from exercising. Stage physically and mentally demanding tasks throughout the day or week. Plan rest periods.
Provide general encouragement (SCogT)	Efforts towards achieving behavior change is rewarded or complimented	For many, it takes several attempts to quit, so keep trying.
Set graded tasks (SCogT)	Set easy tasks for desired behavior change, with incremental levels of difficulty	Start with 10–15 minutes of exercise every other day and gradually increase until you reach your target goal.
Provide instruction (SCogT)	Provide instructions on how to perform a desired health behavior and how to plan for it	It is advised to stand and take a break from your computer, desk or work every 30 minutes.
Model or demonstrate the behavior (SCogT)	A demonstration of how to appropriately perform the desired health behavior is shown by an expert	Adapting your chopping board may assist you in preparing vegetables and onions. This video shows you how: goo.gl/ZNXeQC.
Prompt specific goal setting (CT)	Planning a desired health behavior with an outline on how it can be contextually achieved	There may be benefits for aiming to lower your systolic blood pressure below 130. Discuss your numbers and targets with your doctor.
Prompt review of behavioral goals (CT)	Review and/or re-evaluation of goals or desired health behavior intentions	Improving your diet takes time. Keep a record of how you’re progressing, you’ll be surprised at how far you’ve come.
Prompt self-monitoring of behavior (CT)	Developing strategies to record desired health behavior, such as a keeping diary	Write down 5 good reasons to keep going and to succeed at staying a non-smoker.
Provide feedback on performance (CT)	The person gets feedback on their behavior based on data about desired behavior	If you have so far achieved one of your goals, keep up the good work.
Provide contingent rewards (OC)	Positive feedback such as praise, encouragement, or material rewards, are linked to the achievement of desired behavior	You have been working hard towards the goal of returning to work. You can make it happen!
Teach to use prompts or cues (OC)	Coaching to use environmental cues as reminders to perform a desired health behavior	Drinking a glass of water may be helpful when you feel the urge to smoke.
Agree on behavioral contract (OC)	A written record (contract) of the person’s determination to perform a desired health behavior	I pledge to eat 5 servings of fruit and vegetables every day. Do you agree? Reply Yes or No.
Prompt practice (OC)	Prompt to practice rehearsing the desired behavior	Remember that answering questions in full sentences will improve your speech.
Use follow-up prompts	Contacting the person after intervention is complete	
Provide opportunities for social comparison (SCompT)	Provide opportunities to observe others performing a desired behavior, for example through video or case study	Here is a link to a video of things other stroke survivors found helpful in managing pain: goo.gl/1pHJiJ.
Plan social support or social change (social support theories)	Encourage contemplating on how other people can be of assistance or where/how to access social support systems	Connect with other survivors of stroke through https://enableme.org.au/ and share experiences.
Prompt identification as a role model	Identify opportunities for the person to be a role model to others and influence their behavior	Offer your support to another person with stroke this week at a local support group, or online at enableme.
Prompt self-talk	Foster the use of personal-instruction or encouragement (aloud or silently) to support action	Remember to set a phone reminder to help you take your medications.
Relapse prevention (relapse prevention therapy)	Following initial change, help the person identify and manage situations when there is potential for failure in desired health behavior	When trying to quit smoking, it is best to avoid places where people smoke.
Stress management (stress theories)	Involves specific techniques to reduce anxiety and stress (eg, progressive relaxation) and not necessarily target the desired behavior	Try to do at least 10 minutes of meditation daily.
Motivational interviewing	Involves prompting the person to develop self- motivating statements and evaluations of their own behavior to diminish their resistance to change	Write some motivational statements on sticky pads and put them on your fridge to keep you motivated.
Time management	Helping the person make time and fit the behavior into their everyday routine	Activity burns calories and helps maintain weight. Climbing stairs, parking further away, or walking to the office add up quickly to 30 minutes a day.

**Notes:** Behavior change techniques adapted from work published by Abraham and Michie.^29^ CT is a model of self-regulation whereby a behavior is activated to reduce any perceived discrepancy between a present health condition relative to a point of reference (comparator);^41^ IMB skills model incorporates 3 constructs, including information, motivation and behavioral skills, that are needed to engage in a given health behavior;^42^ OC can be described as a process that attempts to modify behavior through the use of positive and negative reinforcement;^42^ SCogT explains human behavior in terms of a 3-way, dynamic model in which personal factors, environments influences, and behavior continually interact;^43^ TRA recognizes an individual’s intentions and behaviors as being shaped by their own attitude towards a particular behavior, the attitude of others around them, and their perceived control for that behavior;^44^ TPB is guided by beliefs about the consequences of a particular behavior, expectations of other people, and factors that hinder performance of a particular behavior.^45^ The underlying principle of motivational interviewing is to elicit and strengthen an individual’s intrinsic motivation to change by exploring and resolving resistance, and strengthening commitment to change.^46^

**Abbreviations:** CT, Control Theory; IMB, Information-Motivational-Behavioral; iVERVE, Inspiring Virtual Enabled Resources following Vascular Events; OC, Operant Conditioning; TPB, Theory of Planned Behavior; TRA, Theory of Reasoned Action; SCogT, Social Cognitive Theory.

**Table 3:** Independent review of developed health and motivational messages

Independent reviewers’ comments	Number of messages (%)
Original number of health and motivational messages reviewed (a)	1,049
Agreement on messages (including no comment)	671/1,049 (64%)
Disagreement on message content	16/1,049 (2)
Minor changes	316/1,049 (30)
Disagreement on stage(s) of change classification	57/1,049 (5)
Messages deleted following review (b)	5/1,049 (0.5)
Additional messages suggested (c)	73
Additional messages added after review (d)	98
Total messages* ([a − b] + [c + d])	**1,215**

**Notes:**

* Includes only motivational and the health-related messages, and not the administrative messages; each bundle of messages was reviewed by one independent expert.

**Table 4:** Overview of number of messages developed for each potential goal as part of the iVER VE program

Major categories	Goals	Level of disability (as appropriate)^a^	Early message (educational)^b^	Late message (motivational)^b^	Total N
Secondary prevention	Blood pressure control		44	23	67
	Cholesterol		20	16	36
	Physical activity	Moderate	25	8	33
		Slight	62	35	97
	Medication adherence		12	28	40
	Smoking		51	32	83
	Alcohol consumption	General	9	14	23
		Problem	8	5	13
	Weight management	Weight gain	8	7	15
		Weight loss	35	18	53
	Diet/nutrition		49	20	69
Health and body function	Memory, concentration, cognition		32	16	48
	Falls	Slight and moderate	21	5	26
		Moderate	12	5	17
	Urinary incontinence		23	14	37
	Fecal incontinence		16	7	23
	Speech		10	13	23
	Swallowing		10	9	19
	Involvement in decisions		13	16	29
	Mobility	Slight and moderate	7	5	12
		Moderate	6	3	9
	Pain		11	10	21
	Fatigue		20	8	28
	Emotions		18	18	36
	Vision		16	11	27
Activities and participation	Finances		22	12	34
	Return to work		12	9	21
	Spousal relationships		5	4	9
	Friends relationships		7	7	14
	Leisure activities		9	4	13
	Return to driving		6	7	13
	Using public transport		8	5	13
	Household chores (inside)		2	5	7
	Household chores (outside)		3	2	5
	Activities of daily living (dressing, showering)		6	12	18
Environment	Access to information		21	13	34
	Access to health professional and GP		48	14	62
	Face-to-face or online support		6	13	19
General admin messages and instructional					18
General motivational messages			17	52	69
**Total**					**1,233**

**Notes:**

a Where appropriate, messages were categorized to cover a range of disability/functional levels so that they could be tailored to the individual’s level of disability assessed using the modified Rankin scale;^47^

b Messages mapped to the contemplation or preparation Stages of Behavior Change were selected as “early” messages; messages mapped to the action or maintenance Stages of Behavior Change were selected as “late” messages.

**Abbreviations:** iVERVE, Inspiring Virtual Enabled Resources following Vascular Events; GP, General Practitioner.
